# MICAL2 Facilitates Gastric Cancer Cell Migration via MRTF-A-Mediated CDC42 Activation

**DOI:** 10.3389/fmolb.2021.568868

**Published:** 2021-03-24

**Authors:** Yueyuan Wang, Pengxiang Min, Chenxiang Qi, Shuo Zhao, Minjie Yu, Yujie Zhang, Jun Du

**Affiliations:** ^1^Department of Physiology, Nanjing Medical University, Nanjing, China; ^2^The Laboratory Center for Basic Medical Sciences, Nanjing Medical University, Nanjing, China; ^3^The First Clinical Medical College, Nanjing Medical University, Nanjing, China; ^4^Jiangsu Key Lab of Cancer Biomarkers, Prevention and Treatment, Collaborative Innovation Center For Cancer Personalized Medicine, Nanjing Medical University, Nanjing, China

**Keywords:** MICAL2, MRTF-A, migration, gastric cancer, Cdc42

## Abstract

**Aims and Hypothesis:** Cell migration is driven by the reorganization of the actin cytoskeleton. Although MICAL2 is known to mediate the oxidation of actin filaments to regulate F-actin dynamics, relatively few studies have investigated the potential role of MICAL2 during cancer cell migration.

**Methods:** The migratory ability of gastric cancer cells was measured by wound healing and transwell assays. The relationship between MICAL2 expression and MRTF-A nuclear localization was analyzed using gene overexpression and knockdown strategies. The production of reactive oxygen species (ROS) was evaluated by DCFH-DA staining. mRNA and protein levels of MMP9 were measured using qPCR and immunoblotting analysis. The activities of CDC42 and RhoA were assessed using pulldown assays.

**Results:** Depletion of MICAL2 markedly reduced gastric cancer cell migration. Mechanistically, silencing of MICAL2 inhibited the nuclear translocation of MRTF-A in response to EGF and serum stimulation, whereas the contents of MRTF-A remained unchanged. Further analysis showed that silencing of MICAL2 decreased the activation of CDC42 as well as mRNA and protein levels of MMP9. Ectopic expression of MICAL2 augmented MRTF-A levels in the nucleus, and promoted the activation of CDC42, MMP9 expression, and gastric cancer cell migration. Moreover, silencing of MRTF-A inhibited the CDC42 activation induced by overexpression of MICAL2. In addition, MICAL2-induced ROS generation contributed to the effect exerted by MICAL2 on MRTF-A nuclear translocation.

**Conclusion:** Together, these results provide evidence that MICAL2 facilitates gastric cancer cell migration via positive regulation of nuclear translocation of MRTF-A and subsequent CDC42 activation and MMP9 expression.

## Introduction

The first member of the molecule interacting with CasL family (MICAL) was discovered in 2002 (Suzuki et al., 2002). Although there is only one *MICAL* gene in Drosophila, to date, three MICAL genes (*MICAL1*, *2*, and *3*) and two MICAL-like genes (*MICALL1*, *L2*) have been identified in vertebrates (Fremont et al., 2017). The main function of the MICAL proteins is associated with cytoskeleton remodeling and fundamental biological processes such as cytokinetic abscission, vesicle trafficking, axon growth, and cell migration (Giridharan and Caplan, 2014; Fremont et al., 2017). Among these members, MICAL2 is constitutively active in eukaryotic cells (Giridharan et al., 2012). Like other normal human cells, cancer cells use actin remodeling for migration and invasion into surrounding tissue or vasculature during tumor progression and metastasis. However, despite the known role of MICAL2 in actin oxidation (Yoon and Terman, 2018), how MICAL2 influences cancer cell migration remains largely unknown.

MICAL2 is highly expressed in multiple tumors, including gastric cancer, non-small cell lung cancer, and prostate cancer (Ashida et al., 2006; Mariotti et al., 2016). MICAL2-positive cells are found in the invasive front of many cancers, as well as in metastasizing cancer cells inside emboli (Mariotti et al., 2016). Moreover, silencing of MICAL2 promotes mesenchymal to epithelial transition, inhibits the viability as well as the motility and invasive properties of human cancer cells (Mariotti et al., 2016). Recent studies have shown that MICAL2 nuclear export is associated with lung cancer progression (Zhou et al., 2020). Mechanistically, MICAL2 is a flavin-containing monooxygenase and is associated with ROS generation. High levels of ROS can modify the actin cytoskeleton, increase metabolic activity, and function as a second messenger in intracellular signaling cascades to regulate the migratory properties of cancer cells (Storz, 2005; Nourazarian et al., 2014). This suggests that MICAL2 may be a promising therapeutic target against cancer motility; however, the mechanisms underlying the MICAL2-mediated effect on cancer cell migration remain unclear.

Myocardin-related transcription factor A (MRTF-A) is an actin-regulated transcriptional coactivator for serum response factor (SRF). In the resting condition, interaction between globular actin (G-actin) and MRTF-A protein retains MRTF-A within the cytoplasm. Activated RhoA promotes filamentous actin (F-actin) assembly, thereby decreasing the amount of G-actin and leading to the accumulation of MRTF-A in the nucleus, where it associates with SRF to activate target gene transcription and mediate multiple cellular processes. Several stimuli, including TGFB, BMP, and PDGF, are known to activate target gene transcription *via* MRTF-A (Wang et al., 2012; O’Connor and Gomez, 2013; Vasudevan and Soriano, 2014), which in particular associated with the regulation of cytoskeletal dynamics and cell metastasis (Sun et al., 2006; Medjkane et al., 2009). In neurons, MICAL2 was shown to induce depolymerization of nuclear actin, which in turn led to the accumulation of MRTF-A in the nucleus (Lundquist et al., 2014). In the present study, we examined the effects of MICAL2 on MRTF-A nuclear localization and the expressions of migration-related proteins in gastric cancer cells. The results showed that MICAL2 promotes MRTF-A nuclear translocation in a ROS-dependent manner. Furthermore, MICAL2 facilitates gastric cancer cell migration by promoting MRTF-A-dependent activation of cell division control protein 42 homolog (CDC42) and expression of MMP9. Our data establish a novel relationship between MICAL2 and MRTF-A in the context of the regulation of cancer cell motility, that might be involved in facilitating gastric cancer progression.

## Materials and Methods

### Cells and Cell Culture

The human gastric cancer cell lines SGC-7901, BGC-823, MGC-803, and one non-malignant gastric epithelial cell line GES-1 were obtained from the Typical Culture Preservation Commission Cell Bank, Chinese Academy of Sciences. The cells were cultured in Dulbecco’s Modified Eagle’s Medium (DMEM, high glucose) (Thermo Fisher Scientific) supplemented with 10% fetal bovine serum (FBS) (LONSERA) in a humidified incubator at 37°C with 5% CO_2_. Cells were grown on coverslips for immunofluorescence staining and plastic dishes (Corning) for RNA isolation, protein extraction, and wound healing assay. Cells were starved in serum-free medium overnight and then treated with 20 ng/mL EGF (R&D) or 10% serum for the indicated time before harvest. N-acetyl-L-cysteine (NAC), a specific ROS scavenger, was used in this study to deplete ROS levels.

### Plasmids and siRNAs

The GST–RBD fusion (pGEX-4T-2 plasmid) was kindly provided by Dr. Keith Burridge from the University of North Carolina. The MICAL2-containing plasmid (pcDNA3.1-3 × HA-C) and the MRTF-A-containing plasmid (pEGFP-N1) were purchased from Youbio. The siRNAs were synthesized and purified by GenePharma. The sequences of the siRNAs targeting MRTF-A were: #1, 5′-GCUGAAGAGAGCCAGACUATT-3′; #2, 5′-CCACCUCUAUCCUGCACAATT-3′; #3, 5′-CCAGAUGC UGCAGGAGAAATT-3′. The sequence of siRNA targeting MICAL2 was: 5′-GCUGGGAGUUGAAAUCCAUTT-3′.

Cells were grown on plastic dishes until reaching approximately 70–80% confluence, and then transiently transfected with plasmids or siRNA using Lipofectamine 2000 reagent (Invitrogen) according to the manufacturer’s instructions.

### Cell Migration Assay

After transfection with the indicated siRNA or plasmids for 24 h and the cells had reached approximately 95–100% confluence, wounding was performed by scraping through the cell monolayer with a pipette tip. Medium and non-adherent cells were removed, the cells were washed three times with PBS, and then incubated in fresh medium. Cells were permitted to migrate into the cleared area for 24 h. The images were obtained using an inverted microscope (Carl Zeiss) and the change in the wound width was measured. Percentage closure was determined by normalizing difference to width at 0 h.

After transfection with the indicated siRNA or plasmids for 24 h, cells were harvested and suspended in DMEM without FBS. A total of 4 × 10^4^ cells were seeded in the upper chamber of a transwell insert (8 μm pore size). The lower chamber was filled with DMEM containing 10% FBS. After incubation for 24 h, the cells that had migrated to the lower surface were fixed and stained with 0.1% crystal violet. The number of stained cells was counted and images were obtained with a Nikon TS100 microscope.

### MTT Assay

Cell viability was evaluated by a colorimetric assay based on 3-(4,5-dimethylthiazol-2-yl)-2,5-diphenyltetrazolium bromide (MTT) (Sigma-Aldrich). A total of 5 × 10^3^ cells per well were seeded into 96-well plates and transfected with the indicated siRNA. Ten replicates were performed for each group. After culturing for 48 h, the cells were washed and 10 μL of MTT reagent (5 mg/mL) was added. The cells were then incubated in the dark for 2–4 h. Then, excess MTT was removed and the incorporated dye was solubilized in DMSO. The absorbance was measured at 490 nm using a microplate absorbance reader (BioTek Instruments, Inc., Elx800, United States).

### Real-Time Quantitative RT-PCR

Total RNA was prepared using TRIzol reagent (Invitrogen). cDNA was synthesized and qPCR was then performed using the AceQ qPCR SYBR Green Master Mix (Vazyme Biotech Co., Ltd.) on the ABI StepOne^TM^ Real-Time PCR System (Applied Biosystems). The 2^–ΔΔ*CT*^ method was used to calculate gene expression levels using StepOne Software v2.1 (Applied Biosystems). The following primers were used: GAPDH, 5′-CATCAGCAATGCCTCCGCAC-3′ (sense) and 5′-TGAGTCCT TCCACGATACCAAAGTT-3′ (antisense); MICAL2, 5′-CTCAC ACGACACCTGGACCTA-3′ (sense) and 5′-CCACGCTTATC CAATTTGTACCA-3′ (antisense); MRTF-A, 5′-CAAACGGA AGATTCGTTCCCG-3′ (sense) and 5′-TTGAGGTCATCGGC TAGTCTG-3′ (antisense); MMP9, 5′-GGGACGCAGACATCG TCATC-3′ (sense) and 5′-TCGTCATCGTCGAAATGGGC- 3′ (antisense).

### Cytoplasmic and Nuclear Protein Extraction

Cytoplasmic and nuclear proteins were extracted using the Nuclear and Cytoplasmic Protein Extraction Kit (Beyotime). Briefly, cells were harvested by centrifugation and resuspended in cytoplasmic extraction agent A. The solution was vortexed and incubated on ice for 10 min. Cytoplasmic extraction agent B was then added to the cell pellet and the pellet was vortexed and incubated on ice. The supernatant was collected as the cytoplasmic extract. The insoluble fraction was suspended in nuclear extraction agent. After ultrasonication for 30 min, the mixture was centrifuged and the supernatant was collected as the nuclear extract.

### Immunoblotting Analysis

Lysates were prepared and protein concentration was determined using the BCA Protein Assay Kit (Thermo Fisher Scientific). Equal amounts of protein were separated by SDS–PAGE and transferred to pure nitrocellulose membranes. The blots were first blocked and then incubated with the respective primary antibodies overnight, followed by incubation with secondary antibody (Jackson ImmunoResearch, 111-035-003, 1:10000). The following antibodies were used: anti-MICAL2 (Proteintech, 13965-1-AP, 1:1000), anti-GAPDH (Bioworld, AP0066, 1:10000), anti-MMP9 (Bimake, A5725, 1:1000), anti-MRTF-A (CST, 14760, 1:1000), anti-Histone H3 (CST, 4499, 1:1000), anti-RhoA (CST, 2117, 1:1000), anti-CDC42 (CST, 2462, 1:1000). Protein bands were visualized using ECL reagent (Millipore). Digital images of the immunoblots were obtained and analyzed with Quantity One software (Bio-Rad).

### Pulldown Assay

Active RhoA and active CDC42 were pulled down with GST–RBD beads and PAK–CRIB beads, respectively. Briefly, protein lysates were centrifuged, the supernatants were collected into new tubes containing beads precoupled with GST–PBD or PAK–CRIB, and incubated under rotation at 4°C for 30 min. The particles were then solubilized in 25 μL of 2 × SDS loading buffer and subjected to immunoblotting analysis with anti-RhoA or anti-CDC42 antibody.

### Immunofluorescence Microscopy

Cells were fixed in precooled immunostaining fixative and then rinsed three times. After permeabilization in 0.1% TritonX-100, the cells were blocked in 1% BSA, incubated with primary antibody overnight, and then with a FITC-conjugated secondary antibody for 1 h. Cell nuclei were counterstained with DAPI (Southern Biotech). Images were obtained with an Olympus BX51 microscope equipped with an Olympus DP70 digital camera.

### ROS Measurement

Cells on coverslips were stained with 5 μM DCFH-DA (Beyotime) for 20 min at 37°C. After washing three times with PBS, the coverslips were mounted on glass slides. Images were obtained using a fluorescence microscope at an excitation wavelength of 488 nm and at an emission wavelength of 535 nm. ImageJ Software was used here for ROS quantification and value analysis.

### Chromatin-Immunoprecipitation (ChIP)

Chromatin-immunoprecipitation analysis was performed using the Enzymatic Chromatin IP kit (Cell Signaling). In brief, 1.2 × 10^7^ cells were cross-linked to DNA and the nuclear chromatin was digested. Two percent of this chromatin solution was used as input. The remaining solution was divided into three parts and incubated with an anti-Histone-H3 antibody, an anti-IgG antibody, an anti-MRTF-A antibody, respectively, with rotation at 4°C overnight. The next day, Protein G Magnetic Beads were added to each IP reaction. Then, chromatin was eluted, followed by reversal of cross-linking. The MRTF-A/*MMP9* promoter complex signal was measured by PCR. The primer sequences were 5′-CAGGGAGTCTTCCATCACTTT CCCT (sense) and 5′-CCAGCATGAGAAAGGGCTTACACCA-3′ (antisense) for the *MMP9* promoter; and 5′-TACTAGCGGT TTTACGGGCG-3′ (sense) and 5′-TCGAACAGGAGGAGCA GAGAGCGA-3′ (antisense) for GAPDH.

### Statistical Analysis

All experiments were repeated at least three times and whole data are presented as means ± SD. Statistical analysis was performed using GraphPad Prism 8 software. The Student’s *t*-test was used to evaluate the differences between two groups. One-way ANOVA followed by the SNK test was employed for comparisons between more than two groups. *P* < 0.05 represents statistical significance and *P* < 0.01 represents sufficient statistical significance (two-tailed).

## Results

### MRTF-A Facilitates Gastric Cancer Cell Migration

**FIGURE 1:**
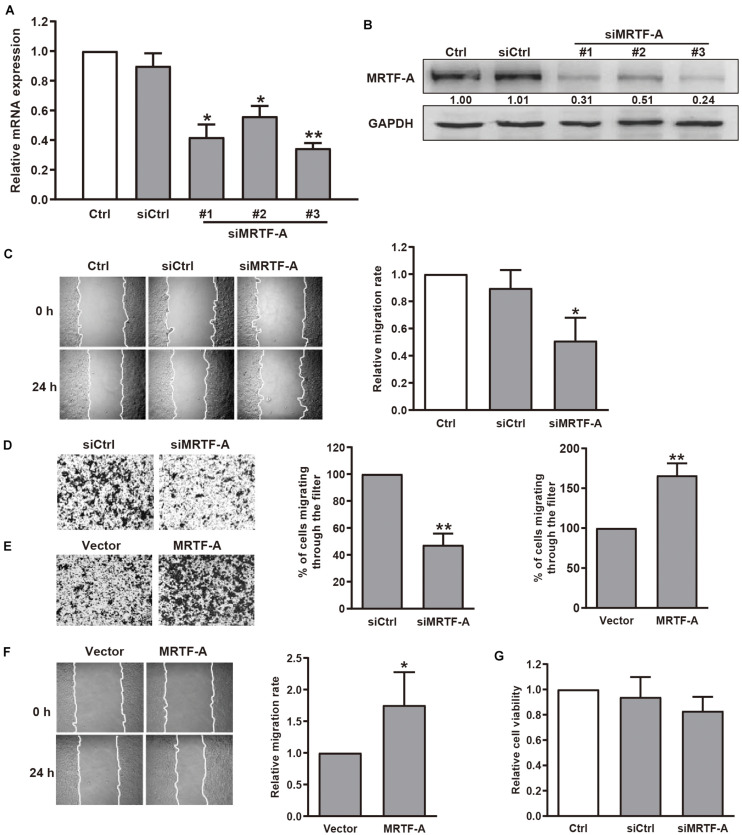
Effect of MRTF-A on gastric cancer cell migration. **(A)** BGC-823 cells were transfected with negative control siRNA or siRNA targeting MRTF-A. After 48 h, *MRTF-A* mRNA expression was analyzed by qPCR. *P < 0.05, **P < 0.01 versus siCtrl group. **(B)** BGC-823 cells were transfected with control siRNA or siMRTF-A, then the protein levels of MRTF-A were examined. Bands corresponding to MRTF-A were quantified and normalized against GAPDH. **(C,D)** The representative of wound healing and transwell migration assays in BGC-823 cells transfected with siMRTF-A are presented, and the quantification of cell migration rate was performed. *P < 0.05, **P < 0.01 versus siCtrl group. **(E,F)** The representative of transwell migration and wound healing assays of MRTF-A-overexpressing cells. *P < 0.05, **P < 0.01 versus vector group. **(G)** The viability of BGC-823 cells transfected with siMRTF-A was detected by MTT assay.

To determine the role of MRTF-A in gastric cancer cell motility, we first detected the protein levels of MRTF-A in several gastric cancer cell lines. Our immunoblotting results indicated that MRTF-A was abundantly expressed in BGC-823 and MGC-803 human gastric cancer cells. Low level of MRTF-A was found in non-malignant gastric epithelial cells GES-1 (Supplementary Figure 1A). Interestingly, MICAL2 expression was also upregulated in BGC-823 and MGC-803 cell lines (Supplementary Figure 1B), BGC-823 and MGC-803 cells were then chosen in the following study. To examine the function of MRTF-A in gastric cancer cell migration, we silenced MRTF-A expression by siRNA transfection in BGC-823 and MGC-803 cells. The cells were lysed and the knockdown efficiency was determined by qPCR (Figure 1A) and immunoblotting assay (Figure 1B and Supplementary Figure 1C). Results of wound healing and transwell migration assays showed that BGC-823 and MGC-803 cells transfected with siRNA targeting MRTF-A exhibited decreased migratory potential compared with control cells (Figures 1C,D and Supplementary Figure 1D). In contrast, an increased migration rate was observed in cells expressing ectopic MRTF-A (Figures 1E,F). The results of MTT assay showed that the viability of BGC-823 cells was not altered after siMRTF-A transfection (Figure 1G), demonstrating that MRTF-A had no effect on cell proliferation. Together, these results indicated that MRTF-A plays a positive role in regulating the migratory potential of gastric cancer cells.

### MICAL2 Induces MRTF-A Nuclear Localization

**FIGURE 2:**
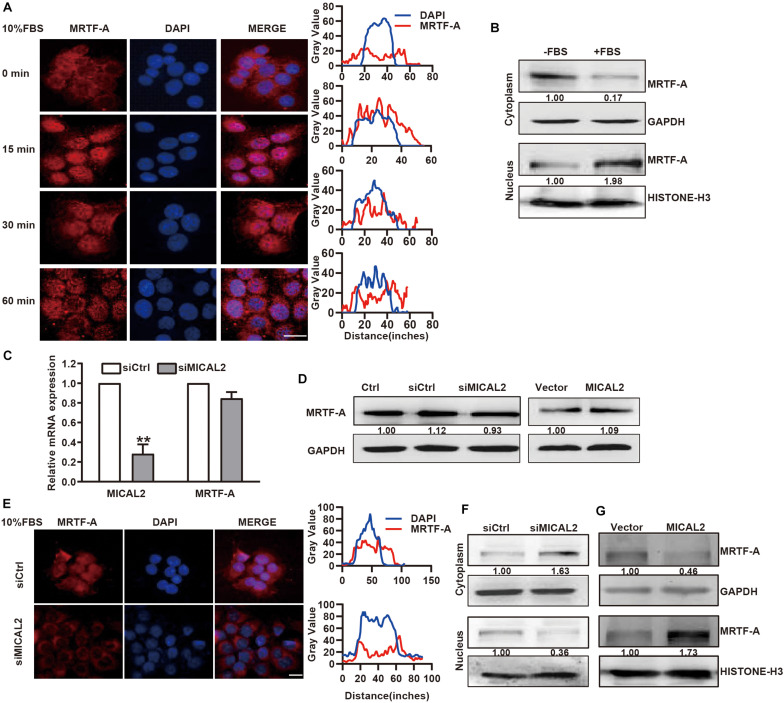
MICAL2 regulates MRTF-A nuclear import. **(A)** BGC-823 cells were in serum-free media overnight and incubated with 10% FBS for the indicated time, then the representative microscopy images of the cells stained for MRTF-A are shown. Scale bar, 10 μm. **(B)** BGC-823 cells were stimulated with 10% FBS for 15 min, and cytoplasmic and nuclear extracts were subjected to immunoblotting analysis to detect the expression of MRTF-A. GAPDH and Histone H3 were used as cytoplasmic and nucleus markers, respectively. **(C)** qPCR analyses of MICAL2 and MRTF-A mRNA in BGC-823 cells which were transfected with siMICAL2. **P < 0.01 versus siCtrl group. **(D)** BGC-823 cells were transfected with siMICAL2 or MICAL2 plasmids, then the protein levels of MRTF-A were examined. **(E)** Representative immunofluorescence images of BGC-823 cells transfected with siMICAL2 and stained for MRTF-A. Scale bar, 10 μm. **(F,G)** BGC-823 cells were transfected with siMICAL2 or MICAL2 plasmids, then the cytoplasmic and nuclear extracts were subjected to immunoblotting analysis to detect MRTF-A expression.

Next, we examined whether the cellular localization of MRTF-A induced by serum could be mediated by MICAL2. Immunofluorescence analysis showed that MRTF-A was weakly expressed in the nucleus of BGC-823 and MGC-803 cells, however, this expression was clearly increased after stimulation with 10% FBS for 15 min (Figure 2A and Supplementary Figure 2A). This result was further confirmed by immunoblotting results (Figure 2B and Supplementary Figure 2B). Then, we transfected cells with siRNA against MICAL2 (Figure 2C) and examined its effect on MRTF-A expression. Knockdown of MICAL2 by siRNA had no significant effect on the mRNA and protein levels of MRTF-A (Figures 2C,D), ruling out the possibility that MICAL2 could regulate nuclear MRTF-A levels by increasing its expression. As shown in Figures 2E,F, siMICAL2-transfected BGC-823 cells exhibited decreased nuclear MRTF-A localization when compared with control cells cultured in DMEM containing 10% FBS. MICAL2-knockdown in MGC-803 cells exhibited similar effects (Supplementary Figure 2C). In contrast, increased nuclear accumulation of MRTF-A was observed in cells overexpressing MICAL2 (Figure 2G). These results indicated that MICAL2 is required for the up regulation of nuclear MRTF-A levels after serum stimulation.

### MICAL2 Promotes MRTF-A Nuclear Localization Through ROS

**FIGURE 3:**
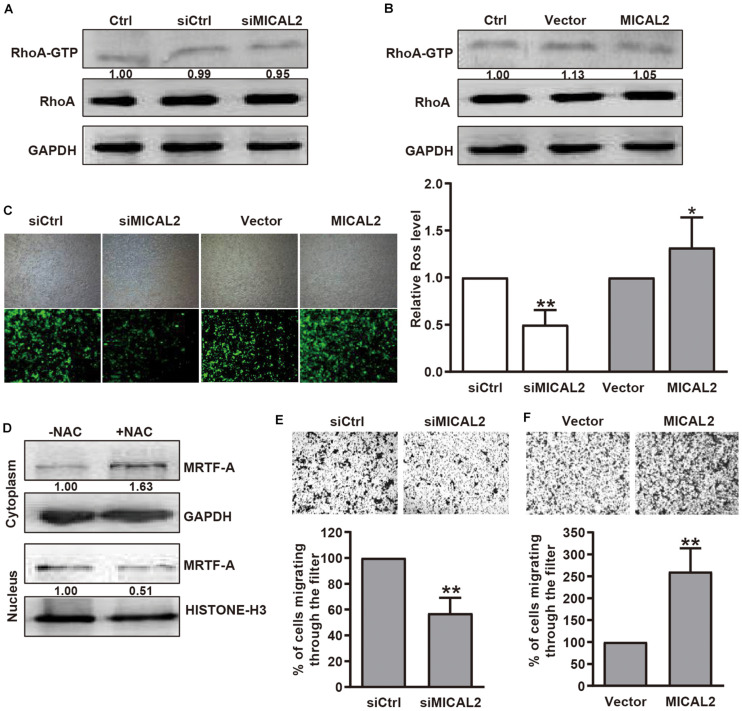
MICAL2 facilitating MRTF-A nuclear import depends on ROS generation, but not RhoA activation. **(A,B)** BGC-823 cells were transfected with siMICAL2 or MICAL2 plasmids, and the activity of RhoA was measured by pull-down assays. **(C)** Effect of MICAL2 on ROS generation. BGC-823 cells were transfected with siMICAL2 or MICAL2 plasmids. Representative micrographs of ROS by DCFH-DA staining are shown. *P < 0.05 versus vector group, **P < 0.01 versus siCtrl group. **(D)** Cells incubated with 2 mM NAC for 1 h, then the extracts of plasma and nuclear section of cells were subjected to immunoblotting analysis to detect the expression of MRTF-A. **(E,F)** Representative images of transwell migration assays in BGC-823 cells transfected with siMICAL2 or MICAL2 plasmids are presented. **P < 0.01 versus siCtrl or vector group.

Previous report showed that SRF/MRTF-A-dependent gene transcription could be induced by RhoA-dependent pathways (Miralles et al., 2003). Since MICAL2 is required for the upregulation of nuclear MRTF-A levels, we considered that MICAL2 might activates SRF/MRTF-A signaling by regulating RhoA activity. However, compared with the control group, no significant change in RhoA activation was found in MICAL2-depleted (Figure 3A) or MICAL2-overexpressing cells (Figure 3B). These data indicated that MICAL2-mediated regulation of cell migration was not dependent on RhoA. As MICAL2 is a monooxygenase and plays a role in ROS generation (Figure 3C and Supplementary Figure 3A), we next treated BGC-823 cells with the ROS scavenger NAC and examined its effect on MRTF-A subcellular location. As shown in Figure 3D, NAC treatment led to a marked decrease in MRTF-A nuclear accumulation, suggesting that MICAL2 may promote MRTF-A nuclear localization through ROS production. As shown in Figures 3E,F and Supplementary Figure 3B, knockdown of MICAL2 reduced the migratory ability of BGC-823 and MGC-803 cells. Meanwhile, the cell migration rate was up regulated by MICAL2 overexpression in those cells. These results suggested that MICAL2 was also required for gastric cancer cell migration.

**FIGURE 4:**
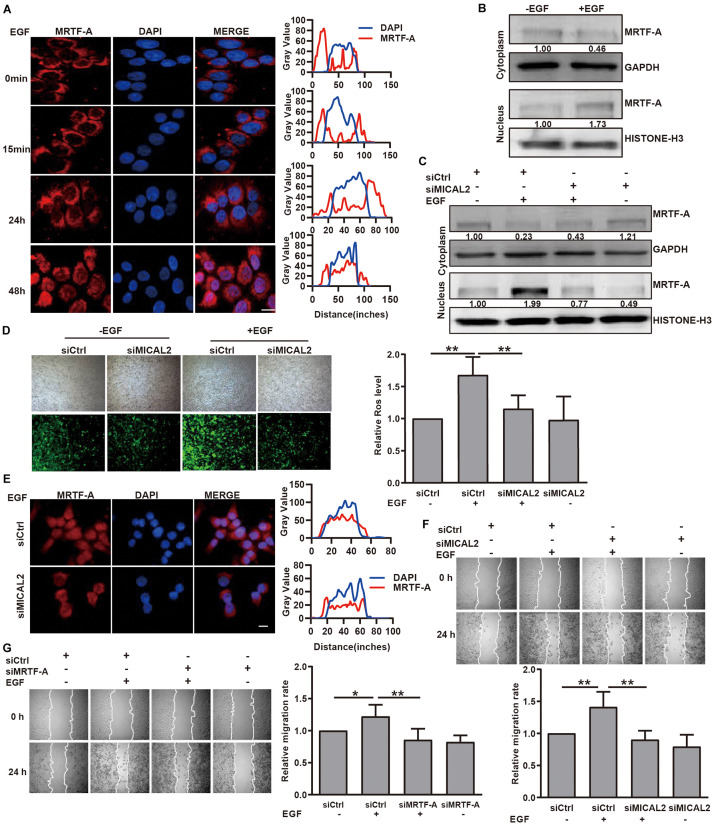
MICAL2 is required for EGF-dependent MRTF-A nuclear import. **(A)** Representative immunofluorescence images of BGC-823 cells incubated with 20 ng/mL EGF and stained for MRTF-A. Scale bar, 10 μm. **(B)** Cells were incubated with EGF for 48 h, then the extracts of plasma and nuclear section of cells were subjected to immunoblotting analysis to detect the expression of MRTF-A. **(C)** Cells transfected with siCtrl or siMICAL2 were incubated by EGF for 48 h, then the extracts of plasma and nuclear section of cells were subjected to immunoblotting analysis to detect the MRTF-A expression. **(D)** Cells transfected with siCtrl or siMICAL2 were incubated in the presence of EGF for 48 h, then were stained with DCFH-DA and taken representative micrographs of those cells. **P < 0.01. **(E)** Cells transfected with siCtrl or siMICAL2 were incubated in the presence of EGF for 48 h, then stained for MRTF-A by immunofluorescence assays. Scale bar, 10 μm. **(F,G)** After transfected with siMICAL2 or siMRTF-A, BGC-823 cells were incubated in the presence of EGF and the migration rate was evaluated by wound healing assays. *P < 0.05, **P < 0.01.

We next examined whether MICAL2 mediates the induction of MRTF-A nuclear import in response to EGF stimulation. Immunofluorescence analysis indicated that EGF stimulation for 48 h led to an increase in MRTF-A accumulation in the nucleus (Figure 4A and Supplementary Figure 4A). This result was further confirmed by immunoblotting results (Figure 4B and Supplementary Figure 4B). Following EGF stimulation, the protein level of MRTF-A in the nucleus was markedly increased in cells transfected with siCtrl, however, the opposite effect was observed following transfection with siMICAL2 (Figure 4C). Meanwhile, the increased ROS generation and MRTF-A nuclear accumulation observed in EGF-cultured cells were also inhibited by siMICAL2 transfection (Figures 4D,E and Supplementary Figure 4C). Furthermore, both siMICAL2 and siMRTF-A transfection suppressed the increased migration rate induced by EGF stimulation (Figures 4F,G). Collectively, these data indicated that ROS generation by MICAL2 promotes EGF-induced MRTF-A nuclear import and gastric cancer cell migration.

### MICAL2 Induces MRTF-A-Dependent CDC42 Activation and MMP9 Expression

**FIGURE 5:**
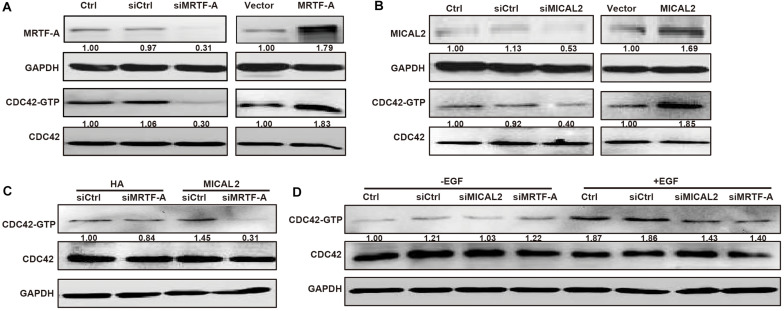
MICAL2 induces MRTF-A-dependent CDC42 activation. **(A)** Cells were transfected with siMRTF-A or MRTF-A plasmids for 48 h, the extracts of cells were subjected to immunoblotting analysis to detect the expression of MRTF-A and activation of Cdc42. **(B)** Cells were transfected with siMICAL2 or MICAL2 plasmids for 48 h, then the extracts of cells were subjected to immunoblotting analysis to detect the expression of MICAL2 and activation of Cdc42. **(C)** Cells were transfected with siMRTF-A plasmids to MICAL2-overexpressed cells, then the extracts of cells were subjected to immunoblotting analysis to detect the activation of Cdc42. **(D)** After incubated with EGF for 48 h, then the activation of Cdc42 in cells transfected with siMRTF-A or siMICAL2 was detected.

According to the analysis of a dataset from The Cancer Genome Atlas (TCGA), *MYH9*, *SRF*, and *VCL*, which are direct target genes of MRTF-A, were all positive correlated with *MICAL2* or *MRTF-A* (Supplementary Figure 5), suggesting a positive correlation might existed between MRTF-A and MICAL2. Rho GTPases are key for the dynamic actin cytoskeletal assembly that forms the basis of cell–cell adhesion and migration. Recently, CDC42, an important Rho GTPase family member, was shown to have an active role in gastric cancer migration and invasion (Du et al., 2016; Liu et al., 2016). Interestingly, we found that knockdown of either MICAL2 or MRTF-A suppressed the activity of CDC42, whereas overexpression of both induced the opposite effect (Figures 5A,B). Meanwhile, siMRTF-A transfection prevented the upregulation of CDC42 activation in MICAL2-overexpressing cells (Figure 5C). Furthermore, both siMICAL2 and siMRTF-A transfection suppressed the EGF-stimulated increase in the levels of activated CDC42 (Figure 5D).

**FIGURE 6:**
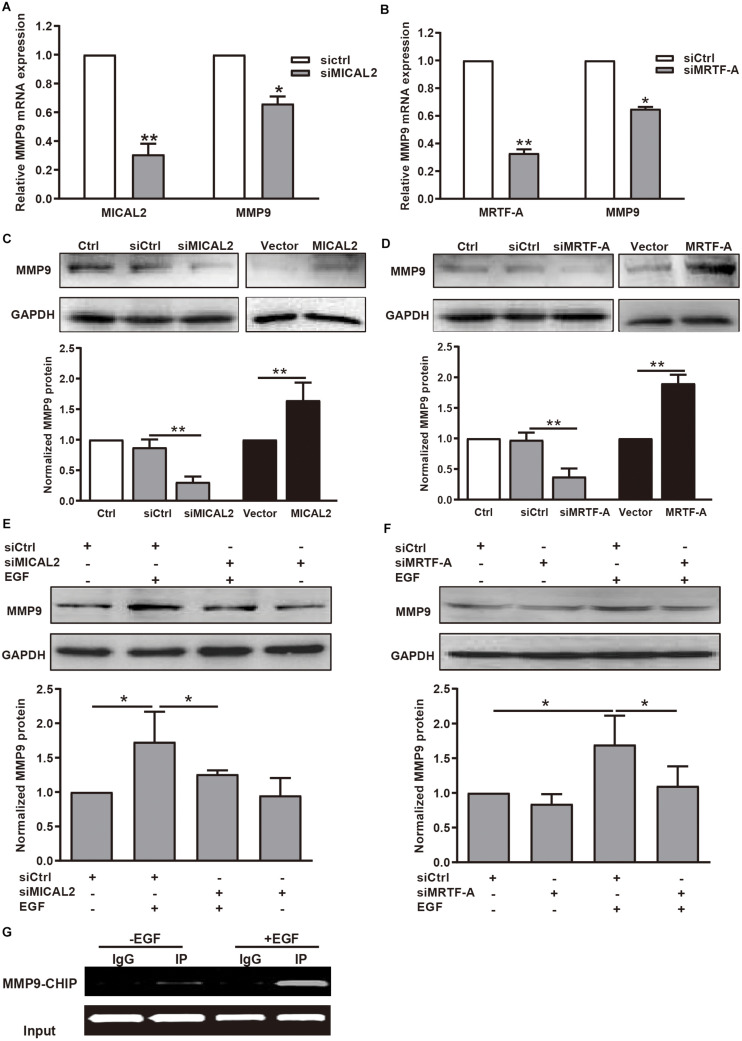
MICAL2 induces transcription of the MRTF-A downstream gene *MMP9*. **(A)** The mRNA levels of MICAL2 and MMP9 in BGC-823 cells transfected with siCtrl or siMICAL2 were detected by qPCR. *P < 0.05, **P < 0.01 versus siCtrl group. **(B)** The mRNA levels of MRTF-A and MMP9 in cells transfected with control siRNA or siMRTF-A. *P < 0.05, **P < 0.01 versus siCtrl group. **(C)** Cells were transfected with siMICAL2 or MICAL2 plasmids for 48 h, then the extracts of cells were subjected to immunoblotting analysis to detect the expression of MMP9. **P < 0.01. **(D)** Cells were transfected with siMRTF-A or MRTF-A plasmids for 48 h, then the extracts of cells were subjected to immunoblotting analysis to detect the expression of MMP9. **P < 0.01. **(E,F)** Cells transfected with siMICAL2 or siMRTF-A were incubated in 20 ng/mL EGF for 48 h, then the extracts of cells were subjected to immunoblotting analysis to detect the expression of MMP9. *P < 0.05. **(G)** Cells were treated with EGF for 48 h, then ChIP analysis was performed to analyze the enrichment of MRTF-A in the *MMP9* promoter region. Normal IgG was used as negative control.

Then we explored whether the mRNA and protein levels of MMP9 were also well correlated with MICAL2 or MRTF-A in gastric cancer cells. We observed that MMP9 transcription was inhibited following transfection with either siMICAL2 or siMRTF-A (Figures 6A,B). Meanwhile, the MMP9 protein level was markedly decreased after knockdown of either MICAL2 or MRTF-A in BGC-823 and MGC-803 cells (Figures 6C,D and Supplementary Figures 6A,B). Overexpression of MICAL2 or MRTF-A elicited the opposite effects. The EGF-induced upregulation of MMP9 protein was also markedly inhibited by transfection of either siMICAL2 or siMRTF-A (Figures 6E,F and Supplementary Figures 6C,D). The occupancy of MRTF-A at the *MMP9* promoter under EGF stimulation was also examined by ChIP assay. Notably, increased occupancy of the *MMP9* promoter by MRTF-A was observed in EGF-treated BGC-823 cells, consistent with the role of EGF in promoting *MMP9* transcription (Figure 6G). These results indicated that MICAL2 may promote gastric cancer cell migration through MRTF-A-dependent CDC42 activation and MMP9 expression.

## Discussion

The nuclear localization of MRTF-A is associated with various signaling pathways involved in cancer progression and metastasis (Scharenberg et al., 2010). Our data demonstrated that MRTF-A depletion impedes EGF- and serum-induced cancer cell migration, which is consistent with previous results implicating *MRTF-A* as an oncogene in leukemia (Ma et al., 2001), pancreatic cancer (Song et al., 2016), and lung cancer (Cheng et al., 2015). Furthermore, we found that both EGF and serum stimulation led to increased nuclear localization of MRTF-A, but did not affect MRTF-A protein contents. The nuclear localization of MRTF-A may result in the transcription of genes containing CArG box regulatory elements (Cao et al., 2011; Luchsinger et al., 2011; Shu et al., 2015). MRTF-A has been shown to upregulate the transcription of genes related to cell migration, including *MYH9*, *SRF*, and *VCL*. The protein expression of MMP9 has been identified as a marker of worse survival and potential metastasis in gastric cancer (Chen et al., 2015). In this study, our results indicated that *MMP9* is also a target gene of MRTF-A. Interestingly, we found that knockdown of either MICAL2 or MRTF-A suppressed the activity of CDC42, whereas this effect was reversed by overexpression of either MICAL2 or MRTF-A. In the GTP-bound conformation, CDC42 could activate PAK, which in turn initiates actin reorganization and cell migration (Guo et al., 2015). Silencing of CDC42 markedly inhibits the migration and invasion of gastric cancer cells (Du et al., 2016). Thus, CDC42 also acts as a critical downstream effector of MICAL2/MRTF-A in cell migration regulation.

In the present study, we showed that EGF- or serum-induced cell migration was greatly diminished after MICAL2 depletion, suggesting that MICAL2 plays a key role in the migration of gastric cancer cells. MICAL2 acts as a physiological mediator of MRTF-A signaling in diverse cell types, including PC12 cells and HEK293T cells, and even *in vivo* zebrafish model (Lundquist et al., 2014). We found that ectopic expression of MICAL2 in gastric cancer cells increased MRTF-A accumulation in the nucleus, which was accompanied by enhanced *MMP9* transcription and CDC42 activation. It is well known that MICAL can oxidize the methionine 44 residue within the D-loop of actin and contribute to F-actin depolymerization (Hung et al., 2011, 2013; Fremont et al., 2017). The ability of MICAL2 to oxidize F-actin shows that MICAL2 can alter the G-actin/F-actin ratio. Indeed, MICAL2 has been shown to regulate G-actin levels and increase MRTF-A accumulation in the nucleus, although the precise mechanisms involved remain to be established (Lundquist et al., 2014). Modulation of cell motility depends not only on actin dynamics in the cytoplasm, but also on the activity of actin in the nucleus (Stern et al., 2009). There seems every reason to believe that MICAL2 might at least partially attribute to the nuclear actin-mediated pathways. Based on our current findings, we propose that MICAL2 may alter the cellular redox status and promote MRTF-A nuclear localization, which is important for regulating the transcription of migration-related genes such as *MMP9*.

To the best of our knowledge, this is the first study to report that MICAL2/MRTF-A is essential for CDC42 activation. CDC42 is a critical regulator of cell migration (Reymond et al., 2012; Razidlo et al., 2018), and activation of CDC42 promotes filopodia and pseudopodia formation, as well as the expression of integrin beta-1, an adhesion receptor known to be involved in metastasis (Wilson et al., 1987). In this study, we found that knockdown of MRTF-A prevented the upregulation of CDC42 activation in the MICAL2-overexpressing cells. We recently reported that MICAL2 potentiates breast cancer cell migration *via* maintaining epithelial growth factor receptor (EGFR) stability, leading to the activation of the P38/HSP27/actin pathway downstream of EGFR (Wang et al., 2018). Here, our data suggested that the MICAL2/MRTF-A complex promotes gastric cancer cell migration through the CDC42 pathway, at least partially. The precise mechanism underlying the MICAL2/MRTF-A-induced activation of CDC42 in gastric cancer cells requires further investigation.

SRF/MRTF-A-dependent gene transcription can be activated by RhoA-dependent pathway, which promotes actin polymerization and leads to the depletion of G-actin in the cytoplasm. RhoA-dependent nuclear translocation of MRTF-A activates the differentiation of mesenchymal stem cells (Jeon et al., 2008) as well as fibrogenesis in human colonic myofibroblasts (Johnson et al., 2014). However, the results showed that knockdown of MICAL2 did not affect RhoA activity. On the contrary, we found that the EGF-induced upregulation of ROS was markedly inhibited by MICAL2 depletion. Meanwhile, the reduction in nuclear MRTF-A levels was observed in response to ROS scavenger NAC pretreatment. Consistent with a recent report showing that RhoA inhibition had no effect on MICAL2-induced activation of the SRF/MRTF-A transcriptional reporter in HEK293T cells (Lundquist et al., 2014), the present data allowing us to conclude that MICAL2 could activate MRTF-A nuclear translocation in a ROS-dependent manner in gastric cancer cells.

## Conclusion

In summary, MICAL2 plays an important role in facilitating gastric cancer cell migration. ROS production by MICAL2, but not RhoA, was identified as a mediator of the nuclear localization of MRTF-A, thereby increasing CDC42 activation and MMP9 expression. These findings establish a novel relationship between MICAL2 and MRTF-A in the regulation of gastric cancer cell motility.

## Data Availability Statement

The original contributions presented in the study are included in the article/Supplementary Material, further inquiries can be directed to the corresponding author.

## Author Contributions

JD designed the study. YW, PM, CQ, and SZ performed the experiments. YW, PM, SZ, MY, and YZ performed the statistical analysis. JD drafted the manuscript. All authors read and approved the final version of the manuscript.

## Conflict of Interest

The authors declare that the research was conducted in the absence of any commercial or financial relationships that could be construed as a potential conflict of interest.

## Supplementary Material

Supplementary Figure 1 Effect of MRTF-A on MGC-803 gastric cancer cell migration. **(A,B)** MRTF-A and MICAL2 protein expressions were detected by immunoblotting analysis in several gastric cancer lines. **(C)** MGC-803 cells transfected with siCtrl or siMRTF-A, and protein levels of MRTF-A were examined. Bands corresponding to MRTF-A were quantified and normalized against GAPDH. **(D)** The representative of wound healing assays in MGC-803 cells transfected with siMRTF-A are presented, and the quantification of cell migration rate was performed. ^∗∗^*P* < 0.01 versus siCtrl group.

Supplementary Figure 2 MICAL2 promotes MRTF-A nuclear import in MGC-803 gastric cancer cells. **(A)** MGC-803 cells were in serum-free media overnight and incubated with 10% FBS for 15 min, and representative microscopy images of the cells staining for MRTF-A are shown. Scale bar, 10 μm. **(B)** MGC-803 cells were stimulated with 10% FBS for 15 min, then the cytoplasmic and nuclear extracts were subjected to immunoblotting analysis to detect the expression of MRTF-A. GAPDH and Histone H3 were used as cytoplasmic and nuclear fraction markers, respectively. **(C)** Representative micrographs of siMICAL2-transfected MGC-803 cells stained for MRTF-A by immunofluorescence staining. Scale bar, 10 μm.

Supplementary Figure 3 ROS mediates MICAL2-induced MRTF-A nuclear import in MGC-803 gastric cancer cells. **(A)** Effect of MICAL2 on ROS generation. MGC-803 cells were transfected with siMICAL2. Representative micrographs of ROS evaluated by DCFH-DA staining are shown. ^∗∗^*P* < 0.01 versus siCtrl group. **(B)** The representative of wound healing assays in cells transfected with siMICAL2 are presented, and the quantification of cell migration rate was performed. ^∗^*P* < 0.05 versus siCtrl group.

Supplementary Figure 4 MICAL2 is required for EGF-dependent MRTF-A nuclear importin MGC-803 gastric cancer cells. **(A)** After incubated with 20 ng/mL EGF for 48 h, representative microscopy images of the cells staining for MRTF-A are shown. Scale bar, 10 μm. **(B)** MGC-803 cells transfected with siCtrl or siMICAL2 were incubated in the presence of 20 ng/mL EGF for 48 h, then the cytoplasmic and nuclear extracts were subjected to immunoblotting analysis to detect the expression of MRTF-A. **(C)** Cells transfected with siCtrl or siMICAL2 were incubated in the presence of EGF for 48 h, then were stained with DCFH-DA and taken representative micrographs of those cells. ^∗∗^*P* < 0.01.

Supplementary Figure 5 The correlation analysis of MRTF-A and MICAL2 with MYH9, SRF, and VCL using The Cancer Genome Atlas (GCTA) database.

Supplementary Figure 6 MICAL2 induces MMP9 expression in MGC-803 gastric cancer cells. **(A,B)** The extracts of siMRTF-A-transfected cells were subjected to immunoblotting analysis to detect the expression of MMP9. **(C,D)** Cells transfected with siMICAL2 or siMRTF-A were incubated in the presence of 20 ng/mL EGF for 48 h, then were subjected to immunoblotting analysis to detect the expression of MMP9.
