# Supplementary figures and images for: MICAL2 Facilitates Gastric Cancer Cell Migration via MRTF-A-Mediated CDC42 Activation
Source: Front Mol Biosci. 2021 Mar 24;8:568868. doi: 10.3389/fmolb.2021.568868 (PMC8024553; doi:10.3389/fmolb.2021.568868)

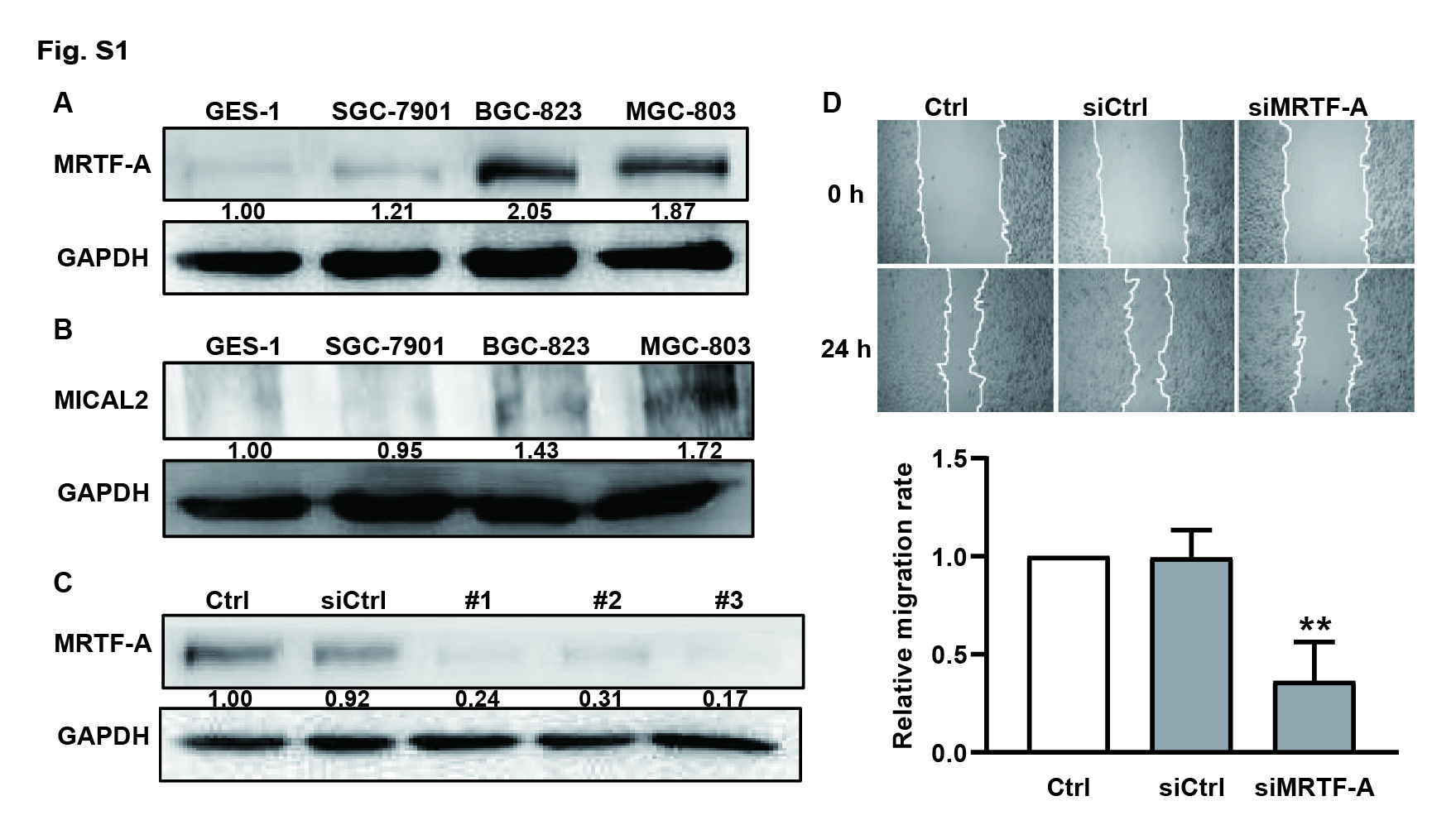

Supplement: Supplementary Figure 1 — Effect of MRTF-A on MGC-803 gastric cancer cell migration. (A,B) MRTF-A and MICAL2 protein expressions were detected by immunoblotting analysis in several gastric cancer lines. (C) MGC-803 cells transfected with siCtrl or siMRTF-A, and protein levels of MRTF-A were examined. Bands corresponding to MRTF-A were quantified and normalized against GAPDH. (D) The representative of wound healing assays in MGC-803 cells transfected with siMRTF-A are presented, and the quantification of cell migration rate was performed. ∗∗P < 0.01 versus siCtrl group. [file Image_1.JPEG]

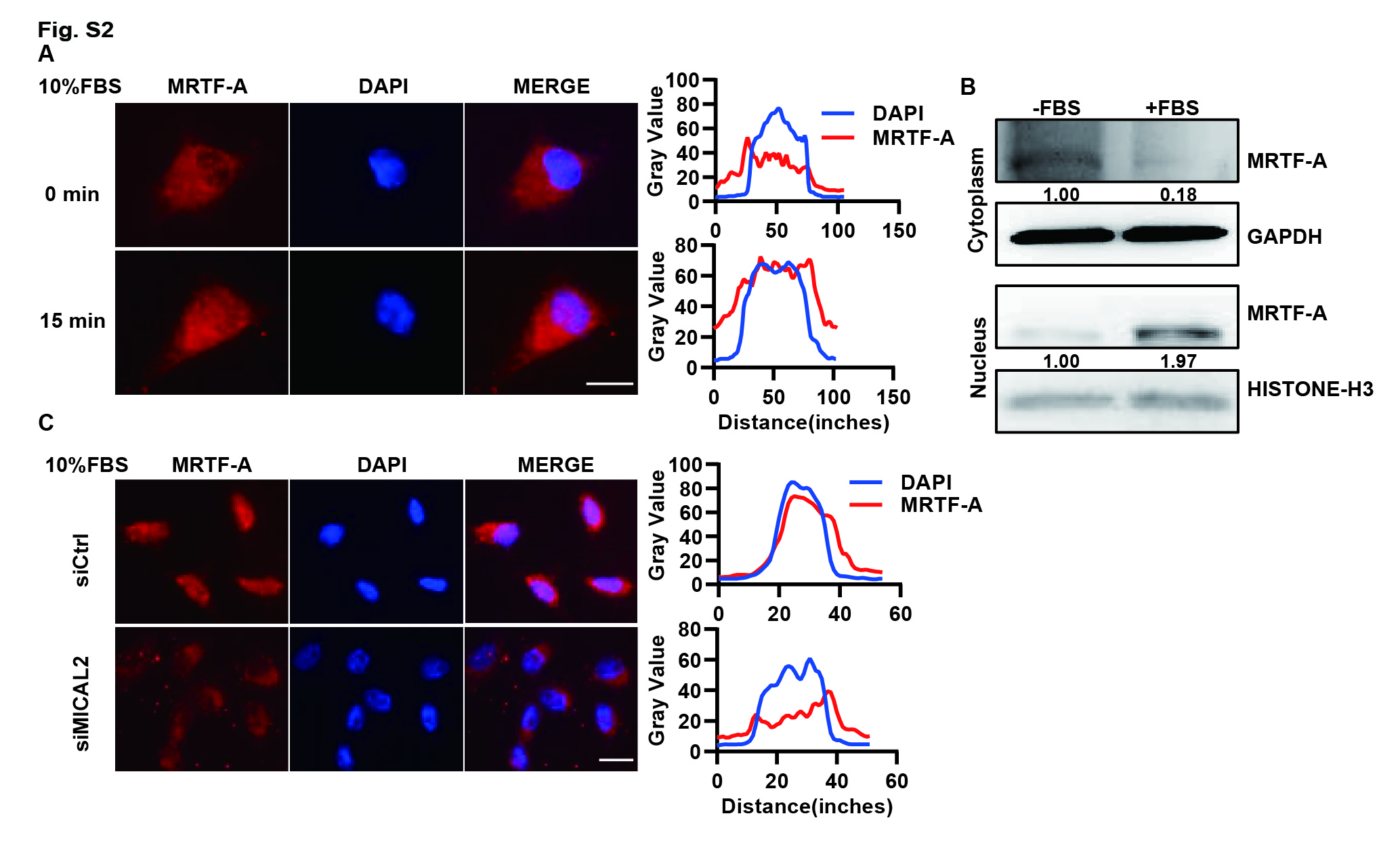

Supplement: Supplementary Figure 2 — MICAL2 promotes MRTF-A nuclear import in MGC-803 gastric cancer cells. (A) MGC-803 cells were in serum-free media overnight and incubated with 10% FBS for 15 min, and representative microscopy images of the cells staining for MRTF-A are shown. Scale bar, 10 μm. (B) MGC-803 cells were stimulated with 10% FBS for 15 min, then the cytoplasmic and nuclear extracts were subjected to immunoblotting analysis to detect the expression of MRTF-A. GAPDH and Histone H3 were used as cytoplasmic and nuclear fraction markers, respectively. (C) Representative micrographs of siMICAL2-transfected MGC-803 cells stained for MRTF-A by immunofluorescence staining. Scale bar, 10 μm. [file Image_2.JPEG]

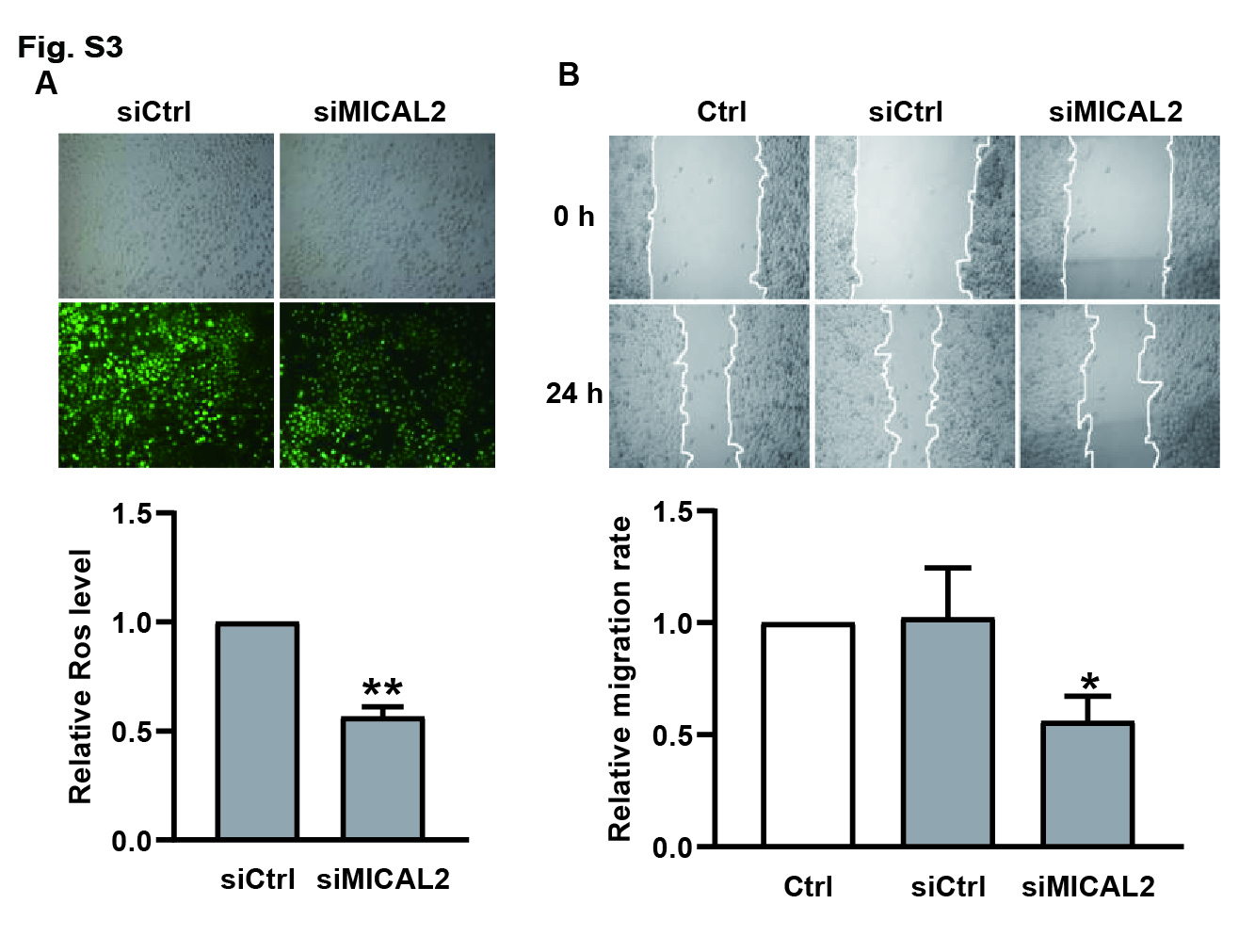

Supplement: Supplementary Figure 3 — ROS mediates MICAL2-induced MRTF-A nuclear import in MGC-803 gastric cancer cells. (A) Effect of MICAL2 on ROS generation. MGC-803 cells were transfected with siMICAL2. Representative micrographs of ROS evaluated by DCFH-DA staining are shown. ∗∗P < 0.01 versus siCtrl group. (B) The representative of wound healing assays in cells transfected with siMICAL2 are presented, and the quantification of cell migration rate was performed. ∗P < 0.05 versus siCtrl group. [file Image_3.JPEG]

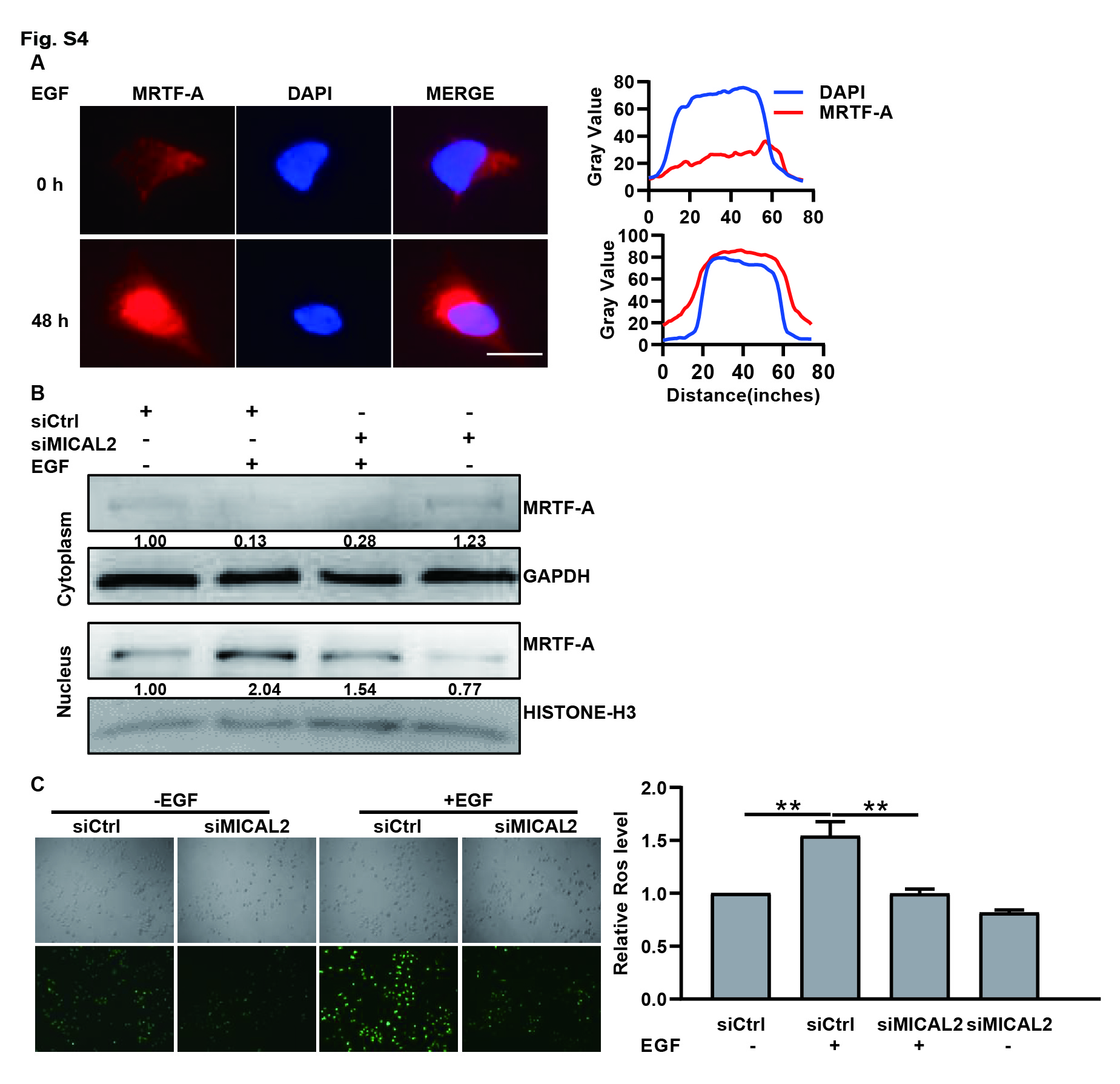

Supplement: Supplementary Figure 4 — MICAL2 is required for EGF-dependent MRTF-A nuclear importin MGC-803 gastric cancer cells. (A) After incubated with 20 ng/mL EGF for 48 h, representative microscopy images of the cells staining for MRTF-A are shown. Scale bar, 10 μm. (B) MGC-803 cells transfected with siCtrl or siMICAL2 were incubated in the presence of 20 ng/mL EGF for 48 h, then the cytoplasmic and nuclear extracts were subjected to immunoblotting analysis to detect the expression of MRTF-A. (C) Cells transfected with siCtrl or siMICAL2 were incubated in the presence of EGF for 48 h, then were stained with DCFH-DA and taken representative micrographs of those cells. ∗∗P < 0.01. [file Image_4.JPEG]

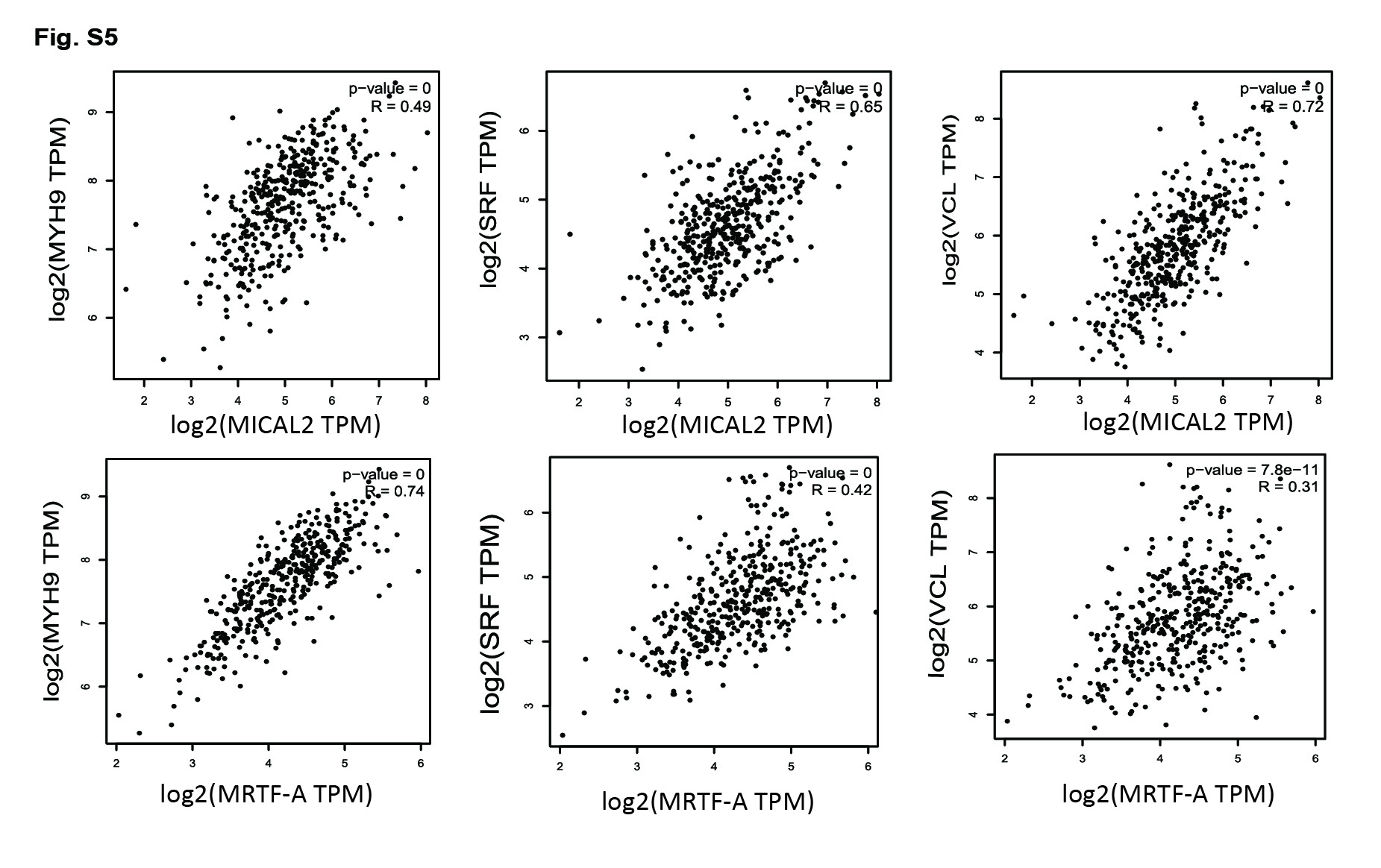

Supplement: Supplementary Figure 5 — The correlation analysis of MRTF-A and MICAL2 with MYH9, SRF, and VCL using The Cancer Genome Atlas (GCTA) database. [file Image_5.JPEG]

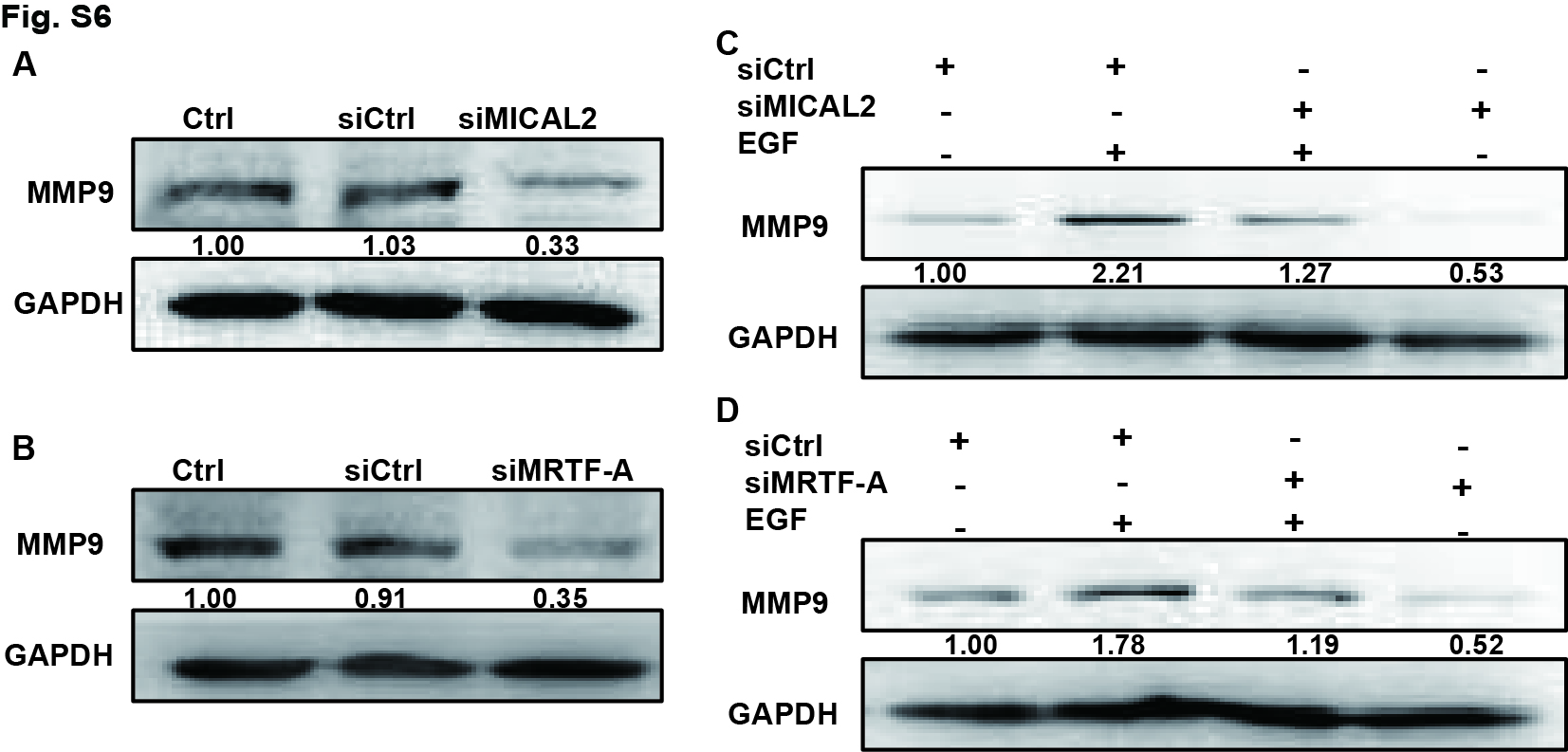

Supplement: Supplementary Figure 6 — MICAL2 induces MMP9 expression in MGC-803 gastric cancer cells. (A,B) The extracts of siMRTF-A-transfected cells were subjected to immunoblotting analysis to detect the expression of MMP9. (C,D) Cells transfected with siMICAL2 or siMRTF-A were incubated in the presence of 20 ng/mL EGF for 48 h, then were subjected to immunoblotting analysis to detect the expression of MMP9. [file Image_6.jpg]
